# Investigating the Effects of a Phytobiotics-Based Product on the Fecal Bacterial Microbiome of Weaned Pigs

**DOI:** 10.3390/ani11071950

**Published:** 2021-06-30

**Authors:** Anlly Fresno Rueda, Ryan Samuel, Benoit St-Pierre

**Affiliations:** Department of Animal Science, South Dakota State University, Brookings, SD 57007, USA; Anlly.Rueda@sdstate.edu (A.F.R.); Ryan.Samuel@sdstate.edu (R.S.)

**Keywords:** phytobiotics, 16S rRNA, swine microbiome, weaning

## Abstract

**Simple Summary:**

Maintaining gut health during the weaning period remains a major challenge for swine producers. Phytobiotics, which are plant-derived bioactive products for use as supplements in food animals, have shown great promise in helping to stabilize the gut environment of weaned pigs, but their mechanisms of action remain unclear. To determine if the positive impact of phytobiotics on gut health may be by influencing the symbiotic bacteria that inhabit the gut of young pigs, this report describes a comparison between fecal bacterial populations of weaned pigs supplemented with a commercial phytobiotic product for seven days to those of untreated pigs. Together, the results indicate that phytobiotic supplementation may provide a favorable environment for both lactate-producing and lactate-utilizing bacteria.

**Abstract:**

The transition to a solid diet, as well as environmental and social stress, have a direct impact on swine gut physiology during weaning, affecting host gastrointestinal functions, as well as resident symbiotic microbial communities. While plant-derived bioactive products, such as phytobiotics, have shown great potential to mitigate these challenges, providing benefits such as antimicrobial, antioxidant, and anti-inflammatory activities, their mechanisms of action remain largely unexplored. To gain more insight, a 21 day trial is conducted to investigate the effects of LiveXtract, a commercial plant-based product, using fecal samples as a proxy for gut bacteria in weaned pigs. High-throughput sequencing of amplicons targeting the V1–V3 region of the 16S rRNA gene is used to determine bacterial composition at days 1 (pre-treatment), 4, 10, and 21 postweaning. Our results show that *Lactobacillaceae* and *Peptostreptococcaceae* are both higher in the supplemented group at D4 (*p* < 0.05), while *Streptococcaceae* are significantly lower in the treated group at D10 and D21. At D10, *Erysipelotrichaceae* are lower, and *Veillonellaceae* are higher in the treated samples than the control group (*p* < 0.05). Of the thirteen abundant Operational Taxonomic Units (OTUs) that have different representation between treated and control pigs (*p* < 0.05), six are predicted to be lactate producers (affiliation to *Lactobacillus* or *Streptococcus*), and one is predicted to be a lactate utilizer, based on its high identity to *Megasphaera elsdenii*. Together, these data suggest that phytobiotics may provide a favorable metabolic equilibrium between lactate production and utilization. Lactate is considered a critical microbial end product in gut environments, as it can inhibit pathogens or be metabolized to propionate for utilization by host cells.

## 1. Introduction

Weaning remains a challenging phase of production for swine operations worldwide. Postweaning diarrhea, typically associated with a high incidence of entero-toxigenic *E. coli* (ETEC) proliferation, causes substantial economic losses resulting in dehydration, growth retardation, and sudden death [1,2,3]. Plant-based extract additives, also known as phytobiotics, have been developed into tools available to the swine industry to mitigate these negative effects [4].

Phytobiotics have been shown to improve animal growth and health, providing benefits, such as antimicrobial, antioxidant, and anti-inflammatory activities, as well as improvement of gut function [5]. However, when considering many different types of chemical and bioactive compounds present in these plant-derived products, the consensus from reported studies is that their modes of action can be highly variable. For example, a combination of essential oils from oregano, anise, and citrus peels was reported to produce similar effects to antibiotics by reducing bacterial colony counts and microbial activity in the gut [6]. On the other hand, incorporation of carvacrol, a phenol present in pepperwort, thyme, and wild bergamot oils, also promoted changes in microbial ecology, but by increasing lactate bacteria, resulting in major changes in gut fermentation [7].

Since the influence of phytobiotics can be attributed not only to a simple decrease or increase in bacterial proliferation, but also to fluctuations in microbiota composition and function [8], further research is required to gain additional insights. In this context, the aim of this study was to evaluate the effects of a plant-based commercial product (LiveXtract; Precision Health Technologies, Brookings, SD, USA) on the diversity and composition of fecal bacterial communities of postweaned pigs. We hypothesized that supplementation with this phytobiotics-based product would affect gut bacterial communities in weaned pigs, and posited that such a study would provide broader insights into a category of additives whose mechanisms of action remain poorly characterized. The inclusion of a phytoextract showed modulation of the fecal microbiome of treated pigs, notably increasing the abundance of candidate lactate-producers and lactate-utilizers.

## 2. Materials and Methods

### 2.1. Animal Management and Sample Collection

Prior to the start of the study, all protocols for the animal trial were approved by the South Dakota State University (SDSU) Institutional Animal Care and Use Committee (IACUC Protocol 18-098A). The animal portion of the study took place at the SDSU Off-Site Wean-to-Finish Barn, a facility that can house up to 1200 pigs and that is managed as a commercial-scale operation. Piglets used in this trial were offspring of PIC females and PIC Duroc-280 boars, and they were weaned at 21 d of age. Upon their arrival at the facility, weaned pigs were randomly assigned to pens independent of any litter information, with equal barrows and gilts distribution. Of the available 48 populated pens in the wean-to-finish barn, two pens of 25 weaned pigs each (6.04 ± 0.15 kg) were used for this study. The same three-phase nursery pig diet was offered in both pens, which followed a standard feeding program for weaned pigs (Phase I [3–4 days], Phase II [5–6 days], Phase III [10–12 days]; Supplementary Table S1). The commercial product used as a source of phytobiotic in this trial, LiveXtract Grazix™, contains plant polyphenols that are extracted by a patented process. The chemical composition of the product and extraction method are both proprietaries. Animals in the treatment pen (Gx) were provided drinking water supplemented with the phytobiotics product at a final dilution of 8 mL per liter of water for seven consecutive days, starting one day after their arrival at the barn. The commercial product is intended to stabilize the gut environment during weaning in young pigs, thus the design of the study was based on the recommended dosage and use by the manufacturer. It was directly added to the water of the treatment pen via a metered chemical injector (HN55; Hydro Systems Company, Cincinnati, OH, USA). The water supplied to the treatment pen runs as a separate waterline from the waterline of the control (Co) pen; both waterlines are supplied by the same county water source. Water was available ad libitum to each pen through two built-in single-nipple cup waterers supplied directly by their respective waterlines. During the phytobiotic treatment period, water for both Gx and Co pens was also supplemented with penicillin G soluble (R-Pen^®^, Huvepharma Inc., Van Buren, AR, USA) at a dose of 250 mg/L, a required preventative measure mandatory for all pigs raised at this facility. Fecal scores were determined at the pen level during the first 11 days of the trial period as an assessment of the overall health of the pigs (Supplementary Table S2). Individual weights were used to select 16 pigs from each pen that were within the same weight range for sample collection (Control pen: 5.1–7.5 Kg; Treatment pen: 5.6–7.3 Kg). Fresh feces were collected from each pig by rectal palpation on postweaning day 1 (before the beginning of treatment), then at days 4, 10, and 21; sample collections were conducted during the same period (09:00–12:00) on the designated days. All fecal samples were stored frozen (−20 °C) until microbial DNA extraction was performed.

### 2.2. Isolation of Microbial DNA and 16S rRNA Gene Amplification by PCR

Total microbial DNA was extracted from fecal samples using a bead beating and column approach [9], which included the QIAamp DNA Mini Kit (Qiagen, Hilden, Germany). Prior to DNA extractions, frozen fecal samples were thawed on ice, from which 250 mg were used for each preparation. The V1-V3 region of the bacterial 16S rRNA gene was amplified by PCR with the Phusion Taq DNA polymerase (Thermo Scientific, Waltham, MA, USA) using the 27F forward [10] and 519R reverse [11] targeting sequences. The oligonucleotides used for amplification consisted of adapters required for MiSeq sequencing fused to the respective sequences of the 27F or 519R primers (adapter sequences underlined): TCGTCGGCAGCGTCAGATGTGTATAAGAGACAGAGAGTTTGATCMTGGCTCAG (Miseq_27F) and GTCTCGTGGGCTCGGAGATGTGTATAAGAGACAGGWATTACCGCGGCKGCTG (MiSeq_519R). Thermal cycling was performed under these conditions: Hot start (4 min, 98 °C), amplification [denaturation (10 s, 98 °C), annealing (30 s, 50 °C) then extension (30 s, 72 °C), for 25 or 35 cycles], final extension (10 min, 72 °C). Template DNA from each sample was first tested with 35 cycles of amplification to confirm the effective production of amplicons of the expected size by agarose gel electrophoresis (~500 bp). Following successful outcomes, templates were amplified under the same protocol for 25 cycles, then PCR reactions were submitted for sequencing with the Illumina MiSeq (2 × 300) platform. Next Generation Sequencing services were provided by the South Dakota State University Genomic Sequencing Facility.

### 2.3. Bacterial Composition Analyses

Unless specified, datasets were analyzed using custom written Perl scripts (available upon request). Overlapping raw forward and reverse MiSeq (2X300) paired-end reads from the same flow cell clusters were assembled into contigs using the ‘make.contigs’ command from MOTHUR (v 1.44) [12]. Assembled 16S rRNA V1–V3 contig sequences were then screened to meet the following criteria: Presence of both intact 27F (forward) and 519R (reverse) primer nucleotide sequences, length between 400 and 580 nt, and an average Phred quality score of at least Q33.

Quality-filtered sequence reads were aligned, then clustered into Operational Taxonomic Units (OTUs). The genetic distance cutoff was set at 4% sequence dissimilarity [13], instead of the more commonly used 3% cutoff for 16S rRNA gene-based analyses. Since 3% as a cutoff was originally recommended for sequences spanning the V1 to V9 variable regions of the 16S rRNA gene, it should be adjusted depending on the sub-region targeted, because variability in sequence dissimilarity is not constant from V1 to V9. Thus, considering that 3% is commonly used for the V4 or V4–V5 regions, which are the least dissimilar of the hypervariable regions, a higher cutoff can be justified for the V1–V3 region, since V1 is the most variable region of the 16S rRNA gene [14]. OTUs generated from clustering were then tested for DNA sequence artifacts by two main strategies. First, screens for chimeric sequences were performed with the commands ‘chimera.uchime’ and ‘chimera.slayer’ from the MOTHUR open source software package [12]. Secondly, the 5′ and 3′ ends of OTUs were tested for the presence of artifacts using a database alignment search-based approach. OTUs were compared to their closest match of equal or longer sequence length, as determined by BLAST (Basic Local Alignment Search Tool) [15] against the NCBI (National Center for Biotechnology Information) ‘nt’ database, and were removed from the analysis if there were more than five nucleotides missing from the 5′ or 3′ end of their respective alignments. OTUs with one or two assigned reads were subjected to an additional screen, where only OTUs with a very close relative in the NCBI ‘nt’ database (alignment spanning the entire sequence of the OTU, with no more than 1% of dissimilar nucleotides) were kept for analysis.

After removal of sequence chimeras and artifacts, RDP Classifier (Ribosomal Database Project) [16] and BLAST [15] were used for taxonomic assignment of valid OTUs. The List of Prokaryotic Names with Standing in Nomenclature (LPSN) was also consulted for information on valid species belonging to taxa of interest [17]. Curated data were rarefied to a sampling depth of 4000 sequences for alpha and beta diversity analyses. Observed OTUs, Chao, Ace, Shannon, and Simpson indices were determined using the ‘summary.single’ command in MOTHUR (v 1.44) [12]. Beta diversity was analyzed by Principal Coordinate analysis (PCoA) based on Bray-Curtis distance, using the ‘Phyloseq’ package of R (v.1.3.959) [18], and visualized using the Tableau Visualization Software (Version 2020.4, Available online: www.tableau.com/products/new-features (accessed on 2 April 2021)).

### 2.4. Statistical Analyses

All statistical analyses were performed in R (Version 3.6.0). Normal distribution of data was first assessed using the Shapiro-Wilk test; alpha diversity indices were found to be normally distributed, while taxa and OTU abundances were not. The Kruskal-Wallis sum-rank test was performed (command ‘kruskal.test’) to determine if the abundances of selected taxa varied across sample groups, and the pairwise Wilcoxon sum-rank test (command ‘pairwise.wilcox.test’) was used to compare abundances between sample group pairs; the Benjamini-Hochberg correction for controlling false discovery rate was applied. Alpha diversity indices were tested using Analysis of variance (ANOVA), with a posthoc ‘HSD.test’ pair-wise function. The ‘adonis’ function from the vegan package [19] was used for permutational multivariate analysis (PERMANOVA, 999 permutations) to detect statistical differences amongst sample sets, followed by the ‘pairwise.adonis’ function from the devtools package to identify pairs of sample groups that were different. For all analyses, tests resulting in *p* ≤ 0.05 were considered significant.

### 2.5. Next Generation Sequencing Data Accessibility

Raw sequence data are available from the NCBI Sequence Read Archive under Bioproject PRJNA721675.

## 3. Results

### 3.1. Pig Growth and Fecal Scores

All control and treatment pigs survived and gained weight during the trial period. No major qualitative differences in fecal scores were observed between pens during the first 11 days of the trial, with only one incidence of diarrhea (observed in the control pen) during this period (Supplementary Table S2).

### 3.2. Taxonomic Composition Analysis of Fecal Bacterial Communities

**Figure 1:**
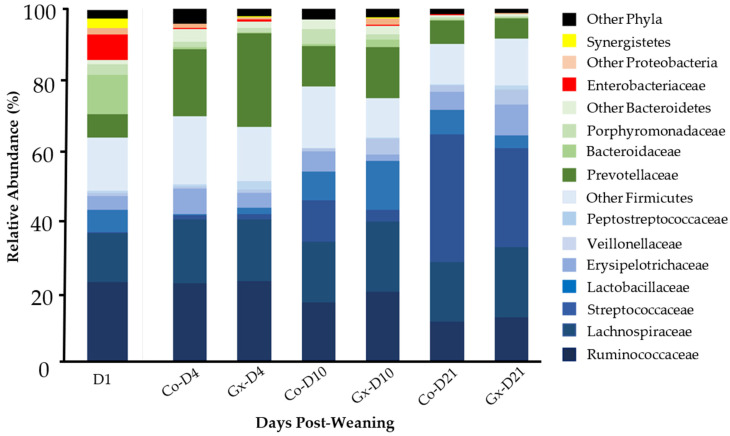
Taxonomic profile at the phylum and family level of fecal bacterial communities of weaned pigs supplemented (Gx) or non-supplemented (Co) with phytoextracts. Families belonging to the same phylum are represented by different shades of the same color: Firmicutes (blue), Bacteroidetes (green), and Proteobacteria (red).

A total of 1,532,519 quality filtered sequence reads were used for the composition analysis described in this report (16,658 ± 8565 reads per sample). Across all experimental groups, four predominant phyla (Firmicutes, Bacteroidetes, Proteobacteria, Synergistetes) were identified. Firmicutes were the most abundant phylum, showing increasing relative abundance from D1 to D21 (Figure 1, Table 1; *p* < 0.05), but no statistically supported differences were found between supplemented (Gx) and non-supplemented (Co) samples at any time point. The most abundant Firmicutes families, Ruminococcaceae and Lachnospiraceae, were not found to be different between treatment groups, with only the former showing a significant reduction over time (Table 1, Figure 1). Notably, other well-represented families from the phylum Firmicutes were found to be significantly different between Gx and Co groups at specific time points (Table 1). Lactobacillaceae and Peptostreptococcaceae, for example, were both lower in the control group at D4 (*p* < 0.05). Conversely, Streptococcaceae were significantly lower in the phytoextract supplemented group at D10 and D21. At D10, Erysipelotrichaceae were higher, and Veillonellaceae were lower in the control samples compared to the treatment group (*p* < 0.05).

Bacteroidetes were the second most abundant phylum across all experimental groups, but, in contrast to Firmicutes, they showed a reduction in abundance between D10 and D21 (*p* < 0.05; Table 1). The three most highly represented families from this phylum showed no significant differences between Gx and Co groups (Table 1); while fluctuations amongst time points were observed, clear patterns could not be resolved using pairwise Wilcoxon comparisons. At D1, Proteobacteria were mainly represented by the Enterobacteriaceae family, displaying a peak level of abundance in contrast to later time points. At D10 and D21, Proteobacteria were significantly higher in the phytoextract supplemented group compared to the control group (Table 1). Synergistetes were abundant at D1, but were then found to be lower by at least 280X by D4, and remained in low abundance at later time points; no differences were detected between Gx and Co samples for this phylum.

### 3.3. OTU Composition Analysis of Fecal Bacterial Communities

**Figure 2:**
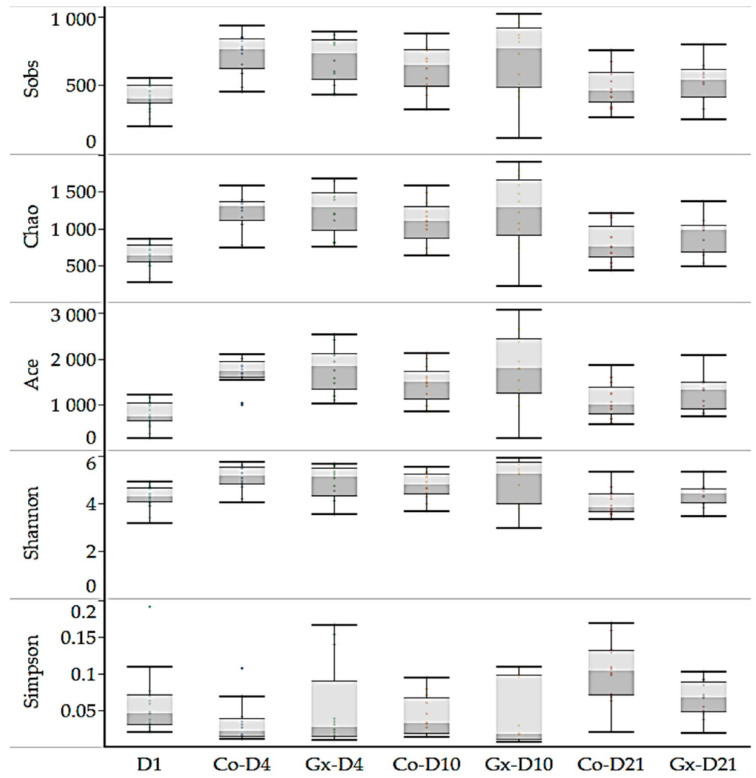
Box plot diagram showing the distribution of alpha diversity indices across the supplemented (Gx) or non-supplemented (Co) groups investigated in this study.

**Figure 3:**
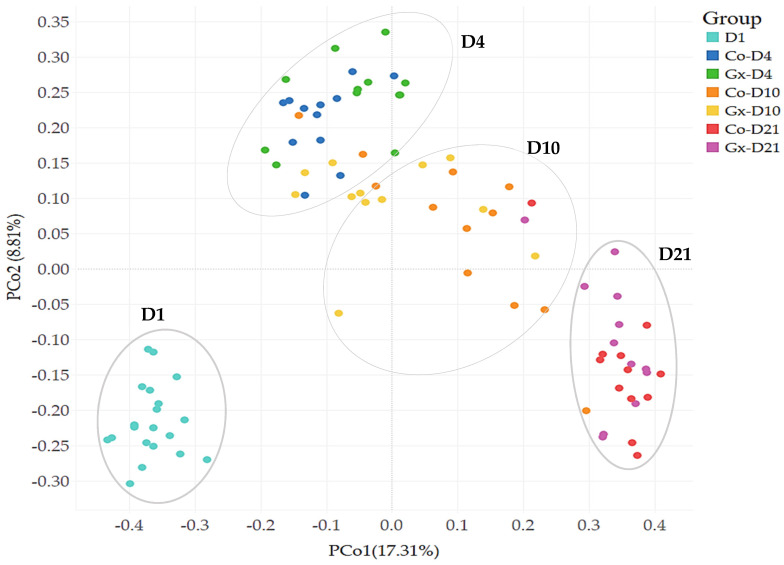
Comparison of fecal bacterial communities between supplemented (Gx) and non-supplemented (Co) samples at different time points (D1, D4, D10, D21). Principal Coordinate Analysis (PCoA) was performed using a Bray-Curtis distance matrix. The x and y axes correspond to Principal Components 1 (PCo1) and 2 (PCo2), which together explain 26.12% of the variance. PERMANOVA and adonis tests indicated differences between groups (*p* = 0.001 for all group comparisons).

A total of 15,996 OTUs were identified across all samples (Supplementary Table S3). Microbial diversity indices showed community level compositional differences amongst fecal bacterial communities across rarefied samples sets, with increases in OTU numbers (observed, Chao, and Ace) between time points D1 and D4/D10 (Table 2, Figure 2), but not between Gx and Co groups at the same time points. Principal Coordinate Analysis (PCoA), PERMANOVA, and pairwise adonis tests showed that samples clustered into four groups (Figure 3). Samples were clustered according to the day of sample collection, with no separation observed between treatment and control samples for D4, D10, or D21 using these methods.

Since PCoA indicated that changes in bacterial composition occurred during the study, further analyses were performed on the most abundant OTUs, which were defined as having a mean relative abundance of at least 1% in at least one group (Table 3). Thirteen of these OTUs were found to differ between Gx and Co groups. At D4, two OTUs (Ssd-00014 and Ssd-00019) were more abundant in samples from supplementation with phytoextract, while the respective levels of five OTUs (Ssd-00001, Ssd-00928, Ssd-00930, Ssd-01244, and Ssd-01332) were found to be lower. Amongst these OTUs, only Ssd-00001 showed a high degree (99.7%) of sequence identity to its closest valid relative, *Lactobacillus amylovorus* (Table 4). Ssd-00331 and Ssd-00039, two OTUs closely related to *Streptococcus alactolyticus* (Table 4), were lower in the phytoextract treatment group at D21 and at both D10 and D21 (*p* < 0.05), respectively. In contrast, OTUs Ssd-00042, Ssd-00071, and Ssd-00188 were lower in the control group at the same time points (*p* < 0.05).

**Figure 4:**
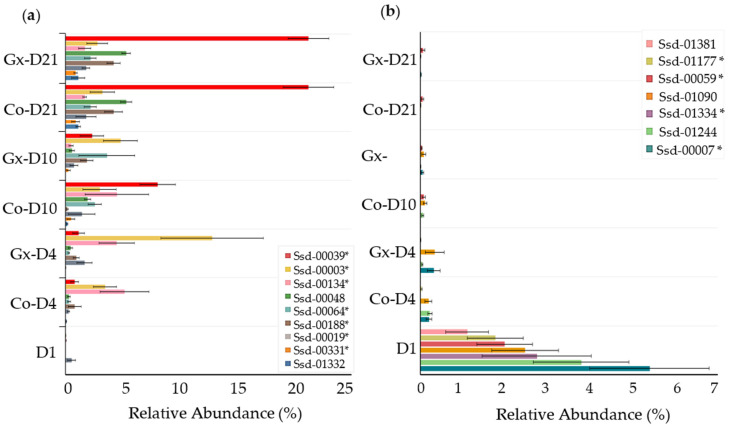
Bar graph comparing the respective abundances of select OTUs across supplemented (Gx) or non-supplemented (Co) groups at different time points. (**a**) OTUs increasing from D1 to D21 (*p* < 0.05). (**b**) OTUs decreasing from D1 to D21 (*p* < 0.05). * OTUs with 97% identity or greater to their respective closest valid taxa.

Other OTUs were found to vary across time points. For instance, nine OTUs that were in low abundance at D1 were notably higher at D21 (Figure 4a). These included Ssd-00039, Ssd-00048 and Ssd-00188, whose levels increased by 2,880X, 531X and 421X, respectively (*p* < 0.05). On the other hand, seven other OTUs displayed an opposite trend with a reduction in abundance over time (Figure 4b; *p* < 0.05). While all OTUs in this category were significantly lower by D4, changes were greater for Ssd-00007, Ssd-01244, and Ssd-01334, with decreases ranging between 282X and 556X (<0.05). Other OTUs showed different abundance fluctuations, such as a peak in abundance at D10 for Ssd-00001, followed by a decrease by D21 (Table 3; *p* < 0.05). Taxonomic affiliation using closest valid species indicated that certain abundant OTUs had a high degree of sequence identity to known bacterial species (five OTUs with 99% or greater, 15 OTUs with 97% or greater), suggesting that they may have represented strains of these species (Table 4).

## 4. Discussion

Environmental and social stress associated with weaning, as well as the transition to a solid diet, have a direct impact on the swine gut microbiota and gastrointestinal tract, resulting in adverse effects on digestive, immune, inflammatory, and barrier functions of the host [20]. This, in turn, reduces the efficiency of growth, and consequently results in lower profitability. Dysbiosis, which is characterized by an imbalance and instability of gut microbial communities [21], is likely to be a common occurrence during the weaning period. In fact, it has been increasingly recognized as the primary cause of postweaning diarrhea, and it is associated with metabolic diseases in weaning pigs [22].

The use of plant-derived products, such as phytobiotics, has become a very attractive option to mitigate the challenges of gut dysbiosis. In fact, a number of phytobiotic-based commercial products have shown promise in animal trials [5,23,24,25,26]. It has been hypothesized that the products include bactericides, which would promote gut microbial community stability by reducing intestinal pathogen pressure [27,28,29]. To gain more insight into the effects of phytobiotics on gut microbial communities, the study described in this report aimed to investigate the effects of a commercial plant-based product on the diversity and composition of gut bacteria from weaning pigs using fecal samples as a proxy.

Observed taxonomic profiles were consistent with previous research studies, as the two most dominant phyla in weaned pig fecal microbiota were Firmicutes and Bacteroidetes [30,31,32]. While the abundance of major phyla did not vary between treatment groups at any given time point, differences were noted at the family and OTU levels, which may indicate gut microbiota modulation due to treatment with phytobiotics. These included bacterial populations affiliated with the family *Lactobacillaceae*, which are generally deemed beneficial for health, with positive effects such as limitation of pathogen growth and improved gut immunity [33,34]. Recent studies have demonstrated similar increases in abundance in response to plant-based products [23], as well as a number of antimicrobial mechanisms expressed by *Lactobacilli* [35,36]. Three of the four prominent *Lactobacillus*-affiliated OTUs were found in higher abundance in samples from pigs supplemented with phytoextract: Ssd-00019, Ssd-00042, and Ssd-00001, which were closely related to *L. reuteri* (98.6%), *L. salivarius* (99.3%), and *L. amylovorus* (99.7%), respectively. As members of the *Lactobacillus* genus, they can be considered important commensals by their synthesis of lactic acid, an end product that can inhibit pathogens [33,37,38,39] or be metabolized to propionate for utilization by host cells [40].

*L. reuteri* has been reported as a common resident of the human and animal gut environments, and is known for utilizing starch [41,42]. Increased abundance of *L. reuteri* with phytoextract supplementation can then be interpreted as a positive effect on young pigs, as these bacterial species have been found to possess probiotic properties, including widespread inhibition of pathogenic bacteria, such as *E. coli* and *Enterobacter*, as well as improvement of feed conversion and live weight gain [43]. Furthermore, *L. reuteri* can produce bacteriocins, suppressing other microbial populations in the gut [42]. *L. salivarius,* on the other hand, ferments complex carbohydrates into D- and L-lactate, which is a combination that can help maintain a balance between microbial utilization and host absorption of bacterial end products [44]. Ssd-00001, which was closely related to *L. amylovorus,* showed fluctuations in abundance across time points, with its highest abundance observed during the transition from phase II to phase III diets. The timing of these changes is consistent with previous studies [32,45], highlighting the impact of diet on modulating gut bacterial composition. *L. amylovorus* has shown potential probiotic properties, including antimicrobial activity against enteric pathogens and maintaining a healthy gut microbiome [46].

Proteobacteria, which include a wide range of pathogens, such as ETEC, was the third most abundant phylum in this study. Its members showed a significant decline after weaning, which is consistent with other reports [30,47,48,49], suggesting that, as the swine gut matures, the abundance of opportunistic pathogens may decrease. Since the respective representation of *Lactobacillus* and Proteobacteria populations appear to have opposite abundance profiles [45], increasing the abundance of members of the *Lactobacillus* genus may have contributed to this reduction. As Proteobacteria have been associated with dysbiosis [50], their declining abundance with host maturity combined with the increased levels of beneficial bacteria in response to phytobiotic supplementation may help in reducing the likelihood of gastrointestinal infections in young pigs.

Recent concerns have been raised regarding lactate metabolism in the animal gastrointestinal tract. While gut bacteria can produce both D- and L-lactate, mammalian cells can only utilize L-lactate enantiomers because they lack the enzymes needed to metabolized D-Lactate [51]. This may result in D-lactate accumulation in the gut, which could lead to acidosis, and be detrimental to the host [52]. Although this metabolic condition is a greater concern in ruminants, where excessive lactate production is typically the result of feeding high amounts of starch-rich ingredients, incidences have also been reported in pigs exposed to stress [53,54]. Regardless of animal species, one common strategy to prevent the onset of acidosis is to favor the activity or higher abundance of D-lactate utilizing gut bacteria that can metabolize this compound into short chain fatty acids (SCFAs). Interestingly, supplementation with phytoextract in this study was associated with increased abundance of *Veillonellaceae,* a family containing D-lactate utilizing species, such as *Megasphaera elsdenii* [55]. Increased abundance of Ssd-00071, which was closely related to *M. elsdenii,* may provide a favorable metabolic equilibrium between lactate production and utilization, reducing the risk of chemical imbalances and metabolic diseases during weaning.

The *Streptococcaceae* family includes a broad range of ecologically significant species that play important roles as part of the normal microbiota that resides in the animal and human gastrointestinal tracts. These functions include lactate production, as well as bacteriocins expression, which provides protection against pathogen proliferation [56]. In this study, three abundant OTUs (Ssd-00039, Ssd-00048, Ssd-00331) were found to be close relatives of *S. alactolyticus.* This bacterial species is a member of the *S. bovis/S. equinus* complex (SBSEC), and has been found to express commensal, pathogenic and food fermenting characteristics [57]. It has been reported in the gut environment of different animals, including pigs, chickens, and dogs [58,59], where it is involved in fermenting saccharides, such as cellobiose, maltose, galactose, fructose, glucose, and mannose into lactate [58,60]. Considering that these substrates are present in most pig diets, these OTUs may correspond to bacterial species that can function as lactate producers in the gut of young pigs. Notably, Ssd-00039 levels in this study were consistent with the report by Poudel et al. [32], where its highest abundance was observed at the end of the phase III diet.

An increase in the abundance of Ssd-00188, a close relative of *Eubacterium rectale* (99.2%), was also observed during the last two weeks of the trial in pigs supplemented with LiveXtract. *E. rectale* is known to metabolize acetate into butyrate, a crucial SCFA for maintaining colonic health in humans and animals [61]. Butyrate is the preferred energy source for colonocytes and colonic mucosa, and it is also important for bacterial energy metabolism [40,62]. Increased abundance of this OTU may represent an additional benefit of phytoextract supplementation in young pigs.

One potential factor that may have affected the outcome of this study was the use of antibiotics in both control and treatment pens. However, antibiotics did not appear to be a confounding factor, as an effect of phytobiotics was still observed. Indeed, OTUs with significantly different representation between the control and treatment groups were identified at each time point. Furthermore, eleven of the most highly represented OTUs in this report have previously been identified as abundant in weaned nursery pigs that were either not exposed to antibiotics [32] or treated with antibiotics using the same protocol as in this study (Poudel et al., unpublished data). While low abundance bacterial species were perhaps affected by antibiotics, this effect would not have been detected since the analysis was focused on the most highly represented OTUs. Possible dose-dependent effects of phytobiotics, as well as their potential interactions with antimicrobials, remain to be determined.

Another observation from this study was PCoA clustering of samples according to the day of sample collection, which was suggestive of microbial succession having occurred during the trial period. These changes in bacterial composition were consistent with the four dietary transitions that took place during this trial (milk, followed by three Phases of nursery diets). Transitions in the bacterial composition of weaned pigs that are associated with changes in diet have previously been reported [32].

## 5. Conclusions

The higher abundance of OTUs closely related to gut bacterial species with probiotic capabilities suggests that LiveXtract supplementation could have a positive impact on the gut microbial balance of young pigs. This, in turn, could help maintain the stability of gut symbiotic bacterial communities, as well as their resistance to perturbations during weaning; thus, mitigating the onset and duration of dysbiotic events. In this context, the results from this study could be used as an initial step towards the design of future trials to test possible mechanical models in a clinical setting.

Of the eleven most highly represented OTUs that have been previously identified as abundant in several weaned nursery pig studies [32,45,63], seven OTUs would be expected to represent novel bacterial species based on their limited sequence identity to cultured or valid species. Further research to investigate their biochemical and metabolic functions in the gut would likely yield valuable insights, notably by helping to determine whether they may represent beneficial gut microorganisms. Given the importance of beneficial gut bacteria during weaning for animal health and nutrition, plant-based products represent tools that could be further developed to improve resistance to pathogens and optimize the use of alternative feed ingredients, as well as provide other benefits to the host animal.

## Figures and Tables

**Table 1:** Mean relative abundance (%) of main bacterial groups in control (Co) and treatment (Gx) samples.

Taxon	D1	Co-D4	Gx D4	Co-D10	Gx D10	Co-D21	Gx D21
Firmicutes #	63.8 ^a^	69.6 ^ab^	66.6 ^ab^	78.1 ^b^	74.8 ^b^	90.1 ^c^	91.5 ^c^
Ruminococcaceae #	22.7 ^a^	22.2 ^a^	22.7 ^a^	16.8 ^ab^	19.6 ^ab^	11.2 ^b^	12.3 ^b^
Lachnospiraceae	13.8	18.0	17.4	17.1	20.0	16.9	20.0
Streptococcaceae #	0.5 ^a^	1.2 ^ab^	1.8 ^b^	11.8 ^c^	3.4 ^b^	36.4 ^d^	28.3 ^e^
Erysipelotrichaceae #	4.0 ^ab^	7.3 ^a^	4.4 ^a^	5.8 ^a^	2.0 ^b^	5.1 ^a^	8.6 ^a^
Lactobacillaceae #	6.2 ^a^	0.3 ^c^	1.6 ^b^	8.2 ^ab^	13.8 ^ab^	7.0 ^a^	3.6 ^a^
Veillonellaceae #	0.9 ^a^	0.5 ^a^	0.9 ^a^	0.7 ^a^	4.4 ^b^	1.6 ^a^	4.4 ^b^
Clostridiaceae1#	0.6 ^a^	7.9 ^b^	7.6 ^b^	6.5 ^bc^	1.2 ^ac^	1.5 ^c^	3.0 ^abc^
Peptostreptococcaceae #	0.6 ^a^	0.7 ^c^	2.2 ^b^	0.2 ^d^	0.2 ^ad^	0.3 ^ad^	1.1 ^abcd^
Eubacteriaceae #	2.9 ^a^	0.2 ^abc^	0.2 ^bc^	0.1 ^c^	0.5 ^abc^	0.6 ^a^	0.4 ^ab^
Clostridiales Inc. Sedis_XIII #	1.0 ^abc^	1.0 ^a^	0.8 ^abc^	1.0 ^a^	1.3 ^ab^	0.4 ^bc^	0.3 ^c^
Other Firmicutes $	10.6	10.4	7.1	10.1	8.5	9.1	9.6
Bacteroidetes #	22.2 ^a^	24.8 ^a^	30.0 ^a^	18.9 ^a^	20.6 ^a^	8.3 ^b^	7.0 ^b^
Porphyromonadaceae #	2.8 ^ab^	1.6 ^ab^	1.2 ^ad^	4.3 ^b^	1.7 ^ab^	0.6 ^cd^	0.4 ^c^
Prevotellaceae #	6.7 ^a^	18.9 ^bc^	26.5 ^b^	11.3 ^ac^	14.4 ^bcd^	6.8 ^ad^	6.0 ^a^
Bacteroidaceae #	11.3 ^a^	0.6 ^bd^	0.5 ^bd^	0.6 ^bd^	2.0 ^b^	0.2 ^cd^	0.2 ^c^
Other Bacteroidetes $	1.36	3.7	1.7	2.7	2.6	0.6	0.5
Proteobacteri #	8.9 ^a^	1.5 ^bd^	1.2 ^b^	0.2 ^cd^	2.1 ^b^	0.2 ^c^	0.4 ^d^
Enterobacteriaceae #	7.1 ^a^	0.3 ^bc^	0.5 ^b^	<0.1 ^de^	<0.1 ^c^	<0.1 ^d^	<0.1 ^ce^
Other Proteobacteria $	1.8	1.2	0.7	0.2	2.0	0.2	0.3
Synergistetes #	2.8 ^a^	0.1 ^b^	<0.1 ^b^	0 *^c^	<0.1 ^c^	<0.1 ^c^	0 *^c^
Other Bacteria $	2.3	4.0	2.2	2.9	2.4	1.6	1.2

# Taxa showing a statistically significant difference by the Kruskal-Wallis sum rank test (*p* < 0.05). Different superscripts in the same row indicate that groups are significantly different by the Wilcoxon pairwise test. $ Statistical test not performed because of group heterogeneity. * no reads detected in any of the samples from this group.

**Table 2:** Analysis of alpha diversity indices across the supplemented (Gx) or non-supplemented (Co) groups investigated in this study. Different superscripts in the same row indicate that groups are significantly different by the Wilcoxon pairwise test.

Index	D1	Co-D4	Gx D4	Co-D10	Gx D10	Co-D21	Gx D21	*p*
Observed OTUs	412 ^a^	722 ^b^	687 ^bc^	620 ^bc^	692 ^b^	485 ^ac^	519 ^abc^	<0.001
Chao	625 ^a^	1217 ^b^	1249 ^b^	1088 ^bc^	1240 ^bc^	798 ^a^	893 ^abc^	<0.001
Ace	789 ^a^	1679 ^b^	1768 ^b^	1448 ^bc^	1772 ^bc^	1089 ^a^	1240 ^abc^	<0.001
Shannon	4.26 ^ab^	5.07 ^c^	4.83 ^ac^	4.74 ^abc^	4.88 ^ac^	4.03 ^b^	4.32 ^abc^	<0.001
Simpson	0.06 ^a^	0.03 ^a^	0.05 ^a^	0.04 ^a^	0.04 ^a^	0.10 ^b^	0.07 ^ab^	0.0016

**Table 3:** Mean relative abundance of the main bacterial OTUs in fecal bacterial communities of weaned pigs supplemented (Gx) or not supplemented (Co) with phytoextracts. Abundance is presented as a percentage (%) of the total number of analyzed reads per sample.

OTUs	D1	Co-D4	Gx D4	Co-D10	Gx D10	Co-D21	Gx D21
Ssd-00001	1.50 ^a^	0.02 ^b^	0 *^c^	4.59 ^ad^	8.09 ^d^	0.95 ^ab^	0.47 ^a^
Ssd-00003	0.04 ^a^	3.45 ^b^	12.84 ^b^	2.98 ^b^	4.80 ^b^	3.23 ^b^	2.78 ^b^
Ssd-00007	5.56 ^a^	0.21 ^b^	0.33 ^b^	<0.01 ^c^	0.05 ^c^	<0.01 ^c^	0.02 ^c^
Ssd-00014	0.04 ^a^	0.44 ^b^	1.48 ^c^	0.11 ^ad^	0.11 ^ad^	0.14 ^d^	0.61 ^bd^
Ssd-00019	0.49 ^a^	0.28 ^a^	1.67 ^bd^	1.42 ^a^	0.73 ^ab^	3.91 ^c^	1.80 ^cd^
Ssd-00039	<0.01 ^a^	0.80 ^b^	1.15 ^b^	8.07 ^c^	2.32 ^b^	28.80 ^d^	21.25 ^e^
Ssd-00042	0.09 ^abcd^	0.03 ^acd^	0.05 ^ac^	<0.01 ^b^	1.88 ^cd^	<0.01 ^d^	0.01 ^abcd^
Ssd-00048	<0.01 ^a^	0.31 ^b^	0.44 ^b^	1.93 ^c^	0.57 ^b^	4.56 ^d^	5.31 ^d^
Ssd-00059	2.04 ^a^	<0.01 ^b^	<0.01 ^b^	0.08 ^c^	0.03 ^c^	0.06 ^bc^	0.07 ^c^
Ssd-00064	<0.01 ^a^	0.32 ^b^	0.29 ^b^	2.57 ^c^	3.62 ^c^	1.80 ^c^	2.17 ^c^
Ssd-00071	0.04 ^a^	0.05 ^a^	0.02 ^a^	0.06 ^a^	1.48 ^b^	0.06 ^a^	1.12 ^b^
Ssd-00134	0.04 ^a^	5.17 ^b^	4.48 ^b^	4.49 ^c^	0.50 ^c^	0.76 ^c^	1.68 ^bc^
Ssd-00188	<0.01 ^a^	0.81 ^bc^	0.94 ^bd^	0.17 ^c^	1.87 ^bd^	2.33 ^d^	4.21 ^e^
Ssd-00331	0 *^a^	0.02 ^b^	0.02 ^b^	0.49 ^c^	0.26 ^bc^	1.90 ^d^	0.89 ^e^
Ssd-00928	0.21 ^ad^	2.06 ^b^	0.55 ^ac^	1.13 ^bc^	0.55 ^ac^	0.10 ^d^	0.03 ^d^
Ssd-00930	4.28 ^a^	1.20 ^a^	0.39 ^b^	0.54 ^ab^	4.82 ^ab^	0.16 ^b^	0.14 ^b^
Ssd-01090	2.54 ^a^	0.20 ^b^	0.35 ^bc^	0.12 ^b^	0.08 ^bcd^	0.01 ^cd^	0.01 ^d^
Ssd-01177	1.82 ^a^	0.03 ^bd^	0.01 ^b^	0 *^c^	<0.01 ^bc^	0 *^c^	<0.01 ^cd^
Ssd-01244	3.90 ^a^	0.23 ^b^	0.05 ^c^	0.06 ^cd^	0.01 ^d^	0 *^e^	0 *^e^
Ssd-01332	0 *^a^	0.08 ^b^	<0.01 ^a^	0.15 ^bc^	<0.01 ^ac^	0.50 ^d^	1.12 ^d^
Ssd-01334	2.82 ^a^	0 *^b^	0 *^b^	<0.01 ^b^	<0.01 ^b^	0 *^b^	0 *^b^
Ssd-01381	1.14 ^a^	<0.01 ^b^	0 *^b^	0 *^b^	<0.01 ^b^	0 *^b^	0 *^b^

All OTUs shown displayed statistically significant differences in abundance (*p* < 0.05) across all seven groups based on the Kruskal-Wallis sum-rank test. Different superscripts in the same row indicate that groups are significantly different by the Wilcoxon pairwise test (*p* values from the Wilcoxon pairwise test are listed in Supplementary Table S4). * No reads detected in any of the samples for this group.

**Table 4:** Closest valid relatives for the main bacterial OTUs that showed a difference in abundance between supplemented (Gx) and non-supplemented (Co) groups (*p* < 0.05). * PI: Percent Identity.

OTUs	Closest Valid Taxon	PI *	Taxonomic Affiliation
Ssd-00001	*Lactobacillus amylovorus*	99.7%	(*Lactobacillaceae)*
Ssd-00003	*Prevotella copri*	97.8%	(*Prevotellaceae)*
Ssd-00007	*Escherichia fergusonii*	98.9%	(*Enterobacteriaceae)*
Ssd-00014	*Terrisporobacter mayombei*	97.3%	(*Peptostreptococcaceae)*
Ssd-00019	*Lactobacillus reuteri*	98.6%	(*Lactobacillaceae)*
Ssd-00039	*Streptococcus alactolyticus*	100%	(*Streptococcaceae)*
Ssd-00042	*Lactobacillus salivarius*	99.3%	(*Lactobacillaceae)*
Ssd-00048	*Streptococcus alactolyticus*	96.5%	(*Streptococcaceae)*
Ssd-00059	*Lactobacillus vaginalis*	98.3%	(*Lactobacillaceae)*
Ssd-00064	*Blautia luti*	97.6%	(*Lachnospiraceae)*
Ssd-00071	*Megasphaera elsdenii*	98%	(*Veillonellaceae)*
Ssd-00134	*Clostridium saccharoperbutylacetonicum*	97%	(*Clostridiaceae)*
Ssd-00188	*Eubacterium rectale*	99.2%	(*Lachnospiraceae)*
Ssd-00331	*Streptococcus alactolyticus*	97.5%	(*Streptococcaceae)*
Ssd-00928	*Ruminococcus gnavus*	95.6%	(*Lachnospiraceae)*
Ssd-00930	*Prevotella stercorea*	96%	(*Prevotellaceae)*
Ssd-01090	*Paludicola psychrotolerans*	88.3%	(*Ruminococcaceae)*
Ssd-01177	*Bacteroides vulgatus*	99.8%	(*Bacteroidaceae)*
Ssd-01244	*Ruminococcus bromii*	91.3%	(*Ruminococcaceae)*
Ssd-01332	*Catenibacterium mitsuokai*	94.5%	(*Erysipelotrichaceae*)
Ssd-01334	*Bacteroides fragilis*	98.7%	(*Bacteroidaceae*)
Ssd-01381	*Enterocloster bolteae*	95.7%	(*Lachnospiraceae*)

## Data Availability

Raw sequence data are available from the NCBI Sequence Read Archive under Bioproject PRJNA721675.

## Supplementary Materials

The following are available online at https://www.mdpi.com/article/10.3390/ani11071950/s1, Table S1: Diet Formulation, Table S2: Pen-level fecal scores in weaned pigs during the first 11 days of the trial, Table S3: Relative abundance of OTUs in each fecal sample from control (Co) weaned pigs or weaned pigs supplemented with a commercial phytobiotic (Gx), Table S4: P values from pairwise comparison of OTUs with the Wilcoxon test (Benjamini-Hochberg correction applied).
