# Supplementary material for: Investigating the Effects of a Phytobiotics-Based Product on the Fecal Bacterial Microbiome of Weaned Pigs
Source: Animals (Basel). 2021 Jun 30;11(7):1950. doi: 10.3390/ani11071950 (PMC8300416; doi:10.3390/ani11071950)
Supplement: Supplementary file 1 [file animals-11-01950-s001.zip › Fresno et al Supplementary Table 1-diets.pdf]

Supplementary Table 1. Diet Formulation (%).

**Ingredient**	**Phase I**	**Phase II**	**Phase III**
Corn,	21.4285	44.315	50.435
Soybean meal,	15	16.765	18.525
Soybean oil,	1.8575	0.779	0.6
Steam rolled oats	20	-	-
Study base mix	-	34.395	16.566
DDGS	-	-	10
Limestone,	1.2	0.5425	1.0205
Whey permeate	20	-	-
Porcine Specialty protein	7.5	-	-
Corn DDG	5	-	-
Soy Protein	5	-	-
Salt,	0.35	0.386	0.547
Monocalcium phosphate 21%	0.515	2.1405	1.368
Biological Protein	0.5	-	-
L-valine,	0.0995	-	-
Lysine HCL,	0.6055	0.3855	0.5785
DL-methionine - 99%,	0.272	0.188	0.1725
L-threonine	0.247	0.0775	0.1385
L-tryptophan	0.05	0.015	0.042
L-isoleucine	0.06	-	-
Other (VTM, etc.)	0.34	-	-
SAM TBCC2[1]	Varied	Varied	Varied
IC TBCC3[2]	Varied	Varied	Varied
TOTAL	100	100	100

[1] SAM Nutrition TBCC at 0.35 lb. provided 100 ppm Cu and at 0.50 lb. provided 150 ppm Cu

[2] IC TBCC at 0.50 lb. provided 150 ppm Cu
