# Supplementary material for: Investigating the Effects of a Phytobiotics-Based Product on the Fecal Bacterial Microbiome of Weaned Pigs
Source: Animals (Basel). 2021 Jun 30;11(7):1950. doi: 10.3390/ani11071950 (PMC8300416; doi:10.3390/ani11071950)

Supplementary Table 2. Pen-level fecal scores in weaned pigs during the first 11 days of the trial.

Treatment pen:

Fecal score	Type 1	Type 2	Type 3	Type 4	Type 5
Day 1	0	0	0	0	0
Day 2	0	+++	+	0	0
Day 3	0	++	+	1	0
Day 4	0	0	+++	0	0
Day 5	0	+++	0	0	0
Day 6	0	+	+++	0	0
Day 7	0	0	++	++	0
Day 9	0	++	++	0	0
Day 11	0	0	+	+++	0

Control pen:

Fecal score	Type 1	Type 2	Type 3	Type 4	Type 5
Day 1	0	0	0	0	0
Day 2	0	0	+++	0	0
Day 3	0	0	++	1	1
Day 4	0	0	+++	0	0
Day 5	0	0	+++	0	0
Day 6	0	0	+++	1	0
Day 7	0	0	++	++	0
Day 9	0	++	++	0	0
Day 11	0	0	++	+++	0

Scale:

0: Fecal type not observed
1: Single instance of fecal type
+: 2 - 4 instances of fecal type
++: 4 - 6 instances of fecal type
+++: 7 or more instances of fecal type

Fecal scoring scale (based on Shu et al., 2006, J Ped Gastroent Nut 33, 171-177)

1

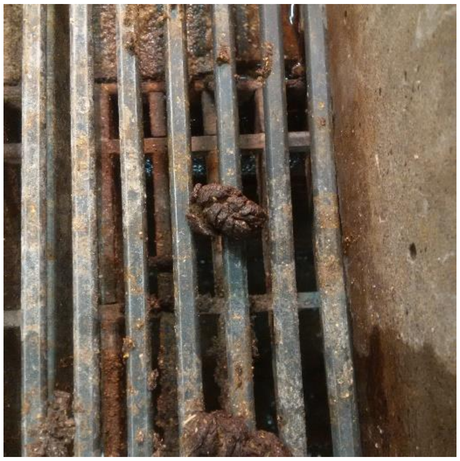

2

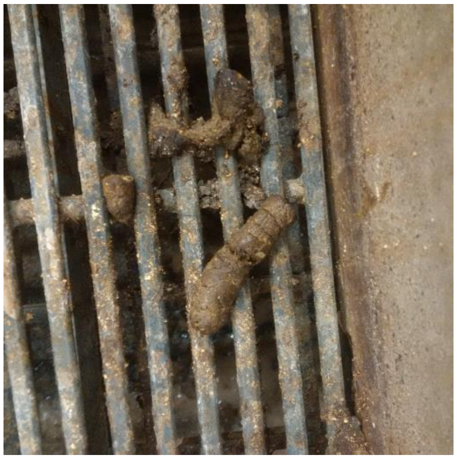

3

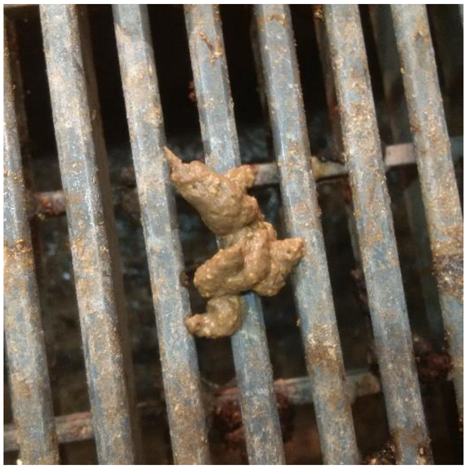

4

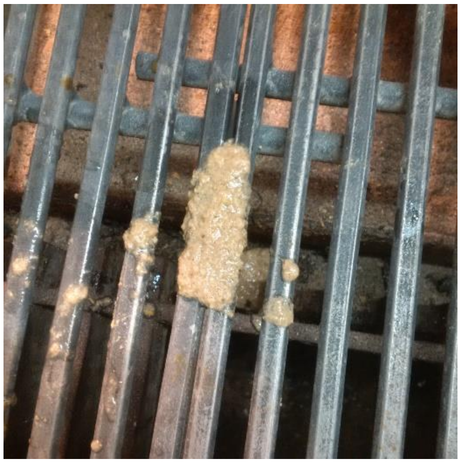

5

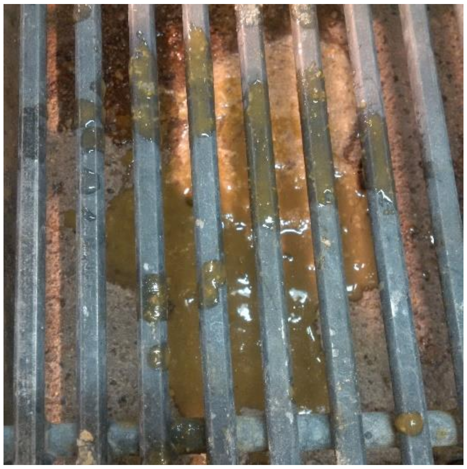

Supplement: Supplementary file 1 [file animals-11-01950-s001.zip › Fresno et al Supplementary Table 2-fecal scores.pdf]
